# Supplementary figures and images for: Cyclin B3 activates the Anaphase-Promoting Complex/Cyclosome in meiosis and mitosis
Source: PLoS Genet. 2020 Nov 2;16(11):e1009184. doi: 10.1371/journal.pgen.1009184 (PMC7660922; doi:10.1371/journal.pgen.1009184)

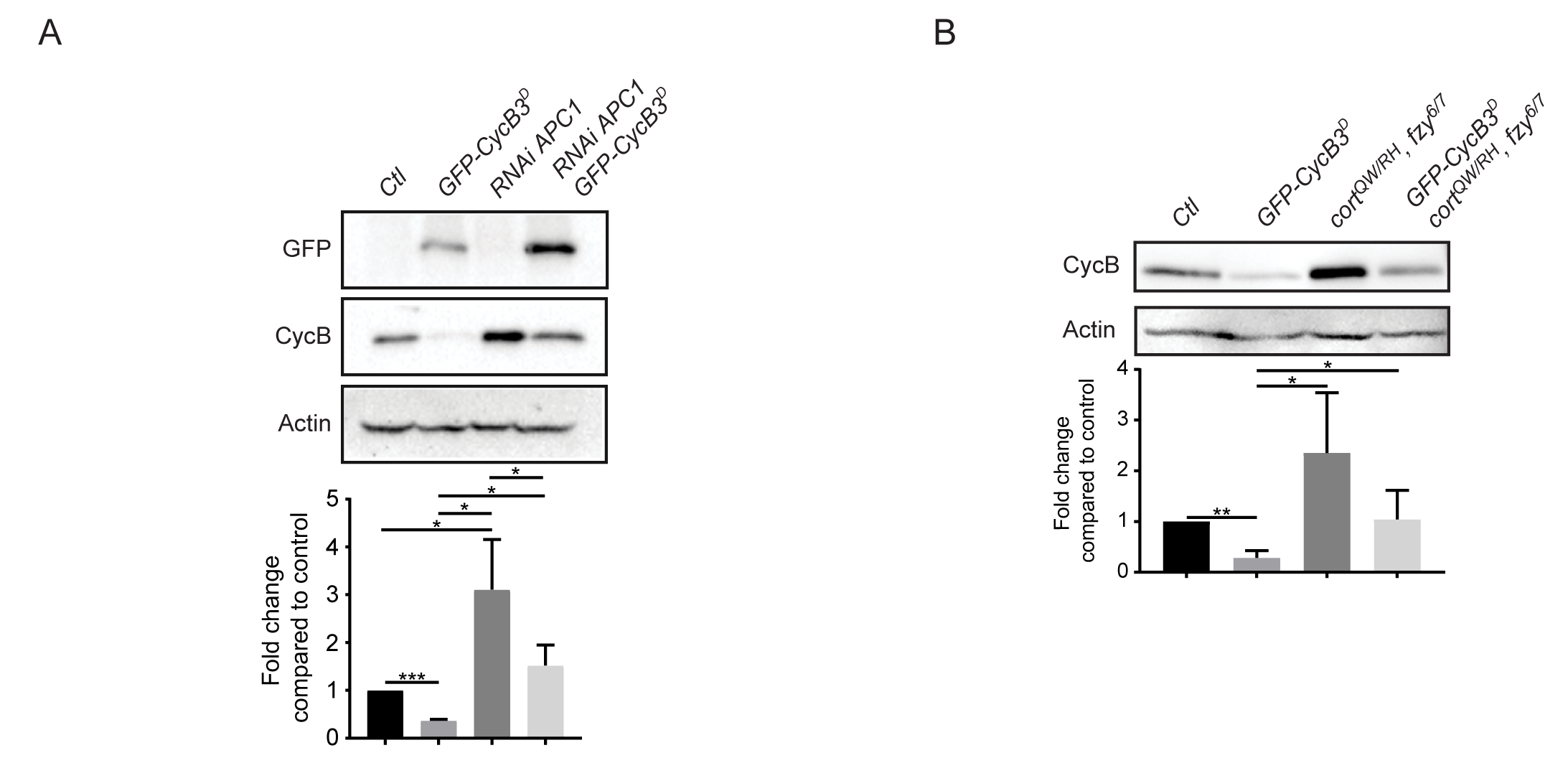

Supplement: S1 Fig — A-B. CycB3 negatively regulates CycB levels in an APC-dependent manner. Eggs were collected for 2 hrs from the indicated conditions and analyzed by Western blot. In A, expression of UASp-GFP-CycB3D and of UASp-APC1 RNAi was driven maternally by Mat α-Tubulin Gal4-VP16. The UASp-GFP-CycB3D and UASp-APC1 RNAi alone genotypes also contained a UASp-WHITE construction, to control for potential dilution of Gal4. Eggs from all conditions failed to develop (Ctl: unfertilized eggs). In B, expression of UASp-GFP-CycB3D was driven maternally by otu-Gal4-VP16. Error bars: SD. **p < 0.01; *p < 0.05 from paired t-tests. Numbers underlying graphs are available in supplemental file S1 Fig Numerical Data. (TIF) [file pgen.1009184.s002.tif]

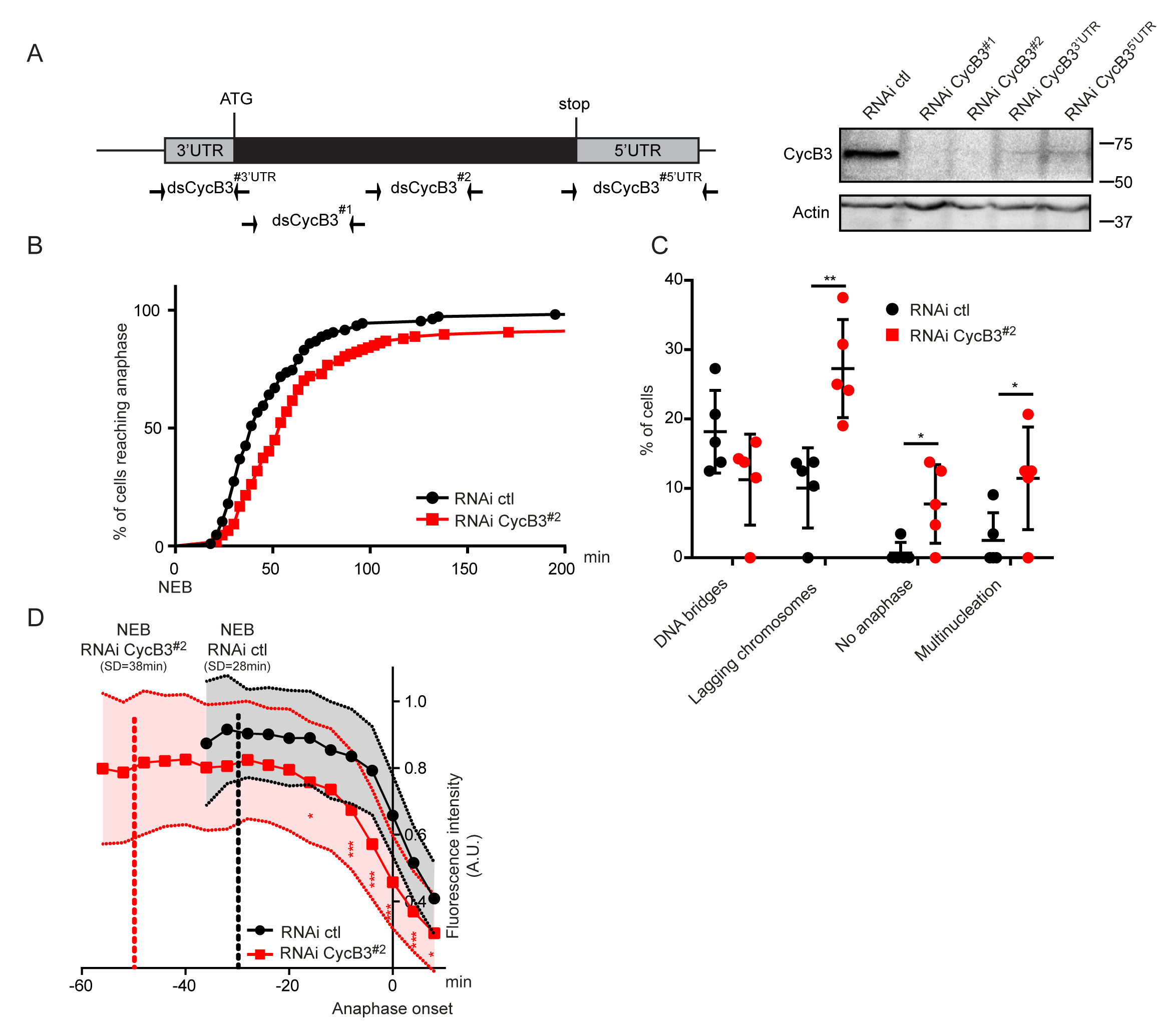

Supplement: S2 Fig — A. dsRNAs targeting different regions of the CycB3 transcript were tested. No 1 and 2 were more effective and selected for experiments. B-D. Similar results were obtained with dsRNA no 2 (here), compared with dsRNA no 1 (Fig 2). B-C. RNAi ctl: 106 cells; RNAi CycB3: 107 cells analyzed. D. RNAi ctl: 30 cells; RNAi CycB3: 40 cells analyzed. Error areas and error bars: SD. ***p < 0.001; **p < 0.01; *p < 0.05 from paired t-tests. Scale bars: 10 μm. Numbers underlying graphs are available in supplemental file S1 Fig Numerical Data. (TIF) [file pgen.1009184.s003.tif]

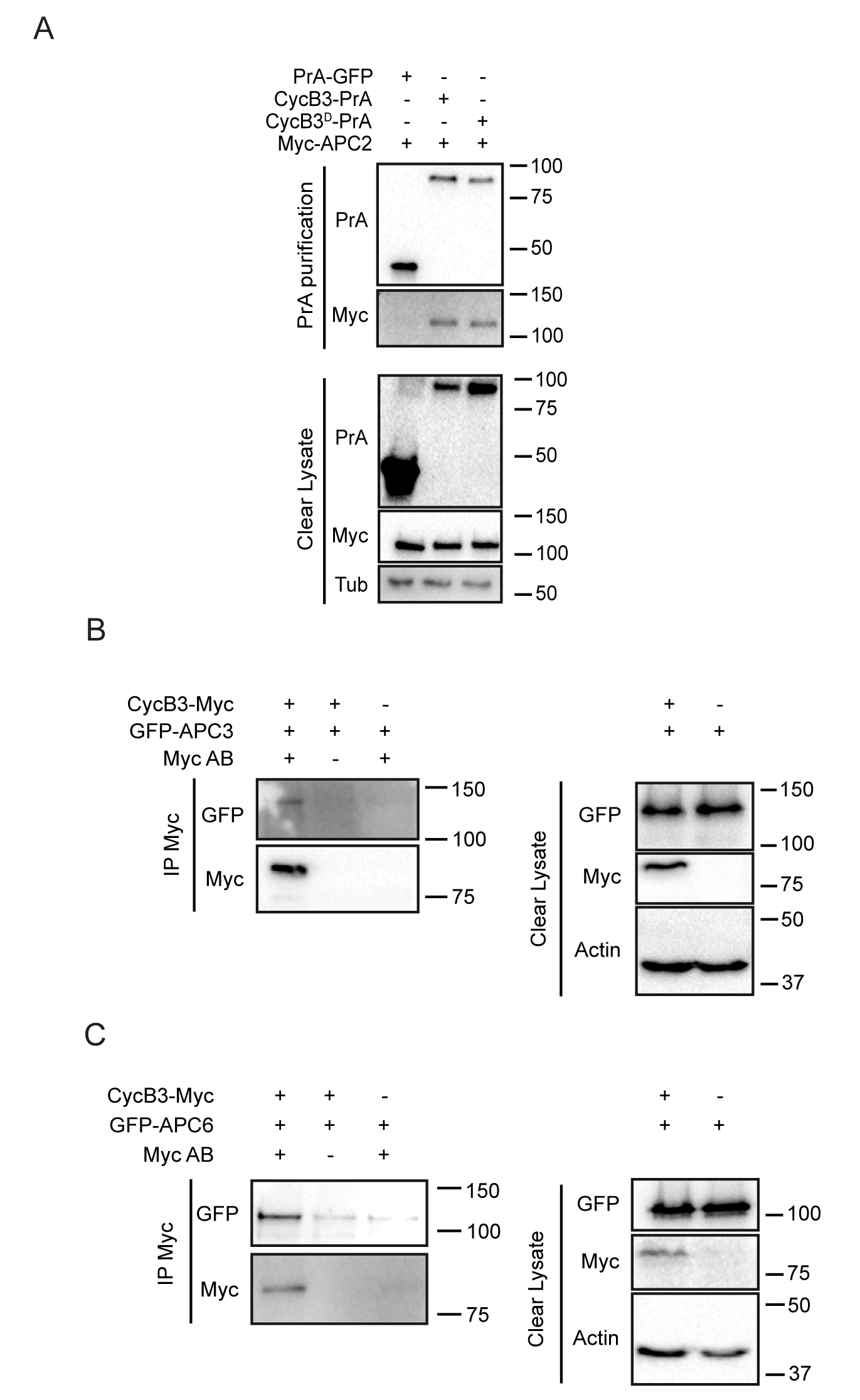

Supplement: S3 Fig — A. Myc-APC2 associates with CycB3-PrA independently from its destruction box. Cells expressing the indicated proteins were submitted to Protein A affinity purification and products were analyzed by Western blot. B-C. CycB3-Myc co-purifies GFP-APC3 (B) or GFP-APC6 (C) in Drosophila syncytial embryos. (TIF) [file pgen.1009184.s004.tif]
